# Impact of financial literacy and education on breast and cervical cancer screening participation in Japan

**DOI:** 10.1371/journal.pone.0313687

**Published:** 2024-11-13

**Authors:** Aliyu Ali Bawalle, Trinh Xuan Thi Nguyen, Mostafa Saidur Rahim Khan, Yoshihiko Kadoya

**Affiliations:** School of Economics, Hiroshima University, Higashi-Hiroshima, Japan; SRM Institute of Science and Technology, INDIA

## Abstract

Despite government efforts, the uptake of screening for breast and cervical cancers among Japanese women remains low. This study employs financial literacy and financial education as proxies for rational decision-making to explore their potential to enhance cancer screening practices in Japan. Using data from Osaka University’s Preference Parameters Study, mean comparison tests and probit regression models are utilized to examine the association between breast and cervical cancer screening and financial literacy and financial education. The results of probit regression show that individuals with higher levels of financial education tend to participate in both breast and cervical cancer screening. In contrast, individuals with higher financial literacy are likely to participate in breast cancer screening, whereas no significant impact is observed for cervical cancer screening. Furthermore, our findings reveal that financial education positively influences both breast and cervical cancer screening. Factors such as employment, marriage, higher education, increased household income, and greater assets demonstrate robust positive relationships with breast and cervical cancer screening. Meanwhile, psychological factors including happiness, a myopic view of the future, anxiety about later life, and perceived health status have no significant associations, except for a positive association between anxiety about life and cervical cancer screening. Our study suggests the development of targeted educational programs that leverage financial literacy and financial education to raise awareness about the importance of breast and cervical cancer screening.

## Introduction

Breast and cervical cancers are two of the most significant health concerns for women worldwide, including in Japan, where they represent a substantial portion of cancer morbidity and mortality. Breast cancer is the most frequently diagnosed cancer among Japanese women, accounting for approximately 21.88% of female cancer cases and 9.68% of cancer-related deaths, making it the fourth leading cause of cancer mortality among women in Japan [1]. The incidence of breast cancer has been steadily rising, partly due to lifestyle changes, increased life expectancy, and better diagnostic practices. Early detection through regular screening is crucial for improving survival rates, as breast cancer, when caught in its early stages, is often more treatable and has a better prognosis [2].

Cervical cancer, while less common than breast cancer, remains a significant public health issue in Japan. In 2023, it was estimated that there were 29,100 new cases of cervical cancer, with 7,200 associated deaths, ranking it fifth in incidence and eighth in mortality among women [3]. Unlike breast cancer, which predominantly affects older women, cervical cancer is often diagnosed in younger women, typically in their 30s and 40s. The primary cause of cervical cancer is persistent infection with high-risk types of human papillomavirus (HPV), which can be effectively prevented through vaccination and regular screening [4]. However, despite the availability of these preventive measures, participation in cervical cancer screening programs remains suboptimal.

Screening for both breast and cervical cancers is recognized as an essential public health strategy to reduce the burden of these diseases. The Japanese government recommends biennial screening for cervical cancer for women aged 20 years and older, and breast cancer screening for women aged 40 years and older [5]. However, the uptake of these screenings is disappointingly low. In 2010, only 24.3% of the eligible population participated in screening programs for breast and cervical cancers, a figure that is significantly lower than the average participation rates in other Organization of Economic Cooperation and Development (OECD) countries [6]. This low participation rate raises concerns about the effectiveness of current public health strategies and highlights the need for innovative approaches to increase screening uptake.

In light of the challenges associated with low participation in female-specific cancer screenings in Japan, this study explores the potential role of financial literacy and financial education as proxies for rational decision-making in addressing this issue. Drawing on Grossman’s rational choice framework [7], which conceptualizes health as a form of capital yielding long-term benefits, we posit that individuals are inclined to invest in their health by engaging in preventive behaviors like cancer screenings. Financial literacy and financial education have been shown in various studies to influence health behaviors by enhancing individuals’ ability to make informed decisions and manage potential future health risks [8–10]. However, irrationality or cognitive limitations can impede these decisions, particularly in the context of cancers, where early detection is critical due to the often late manifestation of symptoms [7,11]. Additionally, emotional factors may also influence these decisions, further complicating the adoption of preventive health measures [12,13].

Existing research supports the idea that financial literacy and education enhance individuals’ ability to make informed economic and health-related decisions, thereby promoting rational health behaviors. Studies have shown that financially literate individuals are more likely to engage in positive health behaviors, such as smoking cessation and regular exercise, due to their improved cognitive abilities and decision-making processes [14–17]. However, while previous research, such as Nguyen et al. [11], has demonstrated a positive association between financial education and general cancer screening uptake, these studies often exclude gender-specific cancers like breast and cervical cancers. This study seeks to address this gap by specifically examining the relationship between financial literacy, financial education, and participation in breast and cervical cancer screenings in Japan.

By investigating these associations, our study aims to provide new insights into how financial knowledge may influence health decisions among Japanese women, potentially informing public health strategies designed to increase participation in cancer screening programs. Our findings align with those of Ling et al. [18], who reported a significant relationship between financial literacy and health investments through enhanced cognitive performance, increased income flows, and enriched health knowledge. Given the global context, where lower financial literacy in developing countries correlates with higher female cancer incidence and mortality [19], our study underscores the importance of promoting financial literacy and education as critical components of both economic and health well-being. We recommend that policymakers prioritize these areas to empower individuals to make rational health decisions, thereby improving cancer screening participation rates.

## Data and method

### Data

We used micro data from the Preference Parameters Study (PPS) of Osaka University’s 21st Century COE Program ‘Behavioral Macrodynamics Based on Surveys and Experiments’ and its Global COE project ‘Human Behavior and Socioeconomic Dynamics’ [20]. The PPS, a panel survey conducted annually from 2003 to 2013, collects information on the socioeconomic and demographic characteristics and preferences of Japanese people. The study employed a multistage sampling and allocation method to collect data throughout Japan, ensuring a comprehensive representation of the population. We used data from the 2010 and 2011 wave of the nationwide survey. The data collection process involved two-stage stratified random sampling. In the first stage, Japan was divided into 10 regional blocks: Hokkaido, Tohoku, Kanto, Koshinetsu, Hokuriku, Tokai, Kinki, Chugoku, Shikoku, and Kyushu. Each region was then further stratified into four categories based on population size: government-designated major cities, cities with populations more than 100,000, cities with populations less than 100,000, and towns and villages. Data collection methods included visits and placement surveys, with responses obtained through face-to-face interviews and other approaches as part of the Preference Parameter Study. This approach ensured that the sample was both geographically and demographically representative of the Japanese population. From the 2010 wave, we use data on financial literacy and financial education as the primary independent variables. Data on the outcome variables and all other explanatory variables are drawn from the 2011 wave. Since the recommended age for cervical cancer screening is 20 years and that for breast cancer screening is 40 years, we target females aged 20 years or over. Combining the two datasets using the panel identification information of each respondent, we exclude data with missing demographic and socioeconomic variables, resulting in 1,729 observations of females 20 years and older, representing 35% of the total responses in the 2011 dataset of 4,934 observations.

### Variables

The dependent variables indicate the probability that an individual had undergone breast and cervical cancer screening the year before the survey. A question in the 2011 PPS dataset asks respondents about their cancer screening behavior: “Over the last 12 months, have you had any cancer examination?"; it has seven response options (stomach cancer, lung cancer, cervical cancer, breast cancer, colon cancer, other cancer examinations, and did not take any cancer examination). We create two binary dependent variables: “1 if the respondent had breast cancer screening and 0 otherwise,” and “1 if the respondent had cervical cancer screening and 0 otherwise.” Focusing on breast and cervical cancers allows us to examine the relationship between female-related cancers and the main explanatory variables.

The main explanatory variables are financial literacy and financial education. Financial literacy is measured using three relevant questions—a method originally developed by Lusardi and Mitchell [14] and subsequently replicated by various authors across different fields [15–17]. For clarity, the specific questions used in this study are detailed in Appendix A. These questions assess basic financial concepts such as interest rates, inflation, and risk diversification, which are essential for evaluating an individual’s financial literacy. For financial education, respondents are asked in the PPS survey: “Did you receive any compulsory financial education when you were in elementary school?” A value of 1 is assigned if the respondent answered “yes” and 0 if they responded “no” or “do not know.” Various socioeconomic and demographic variables such as income, education, unemployment, and risky health behaviors are included as explanatory variables. These indicators enhance our understanding of the multifaceted dynamics influencing female cancer screening [21–23]. Additionally, variables such as happiness, anxiety about later life, future myopic views, and health status are considered. Table 1 summarizes the definitions of the variables and their coding methods.

**Table 1:** Variables definition.

Variables	Definitions
Breast Cancer	1 if respondents select breast cancer screening from the seven options provided in the survey question, and 0 otherwise
Cervical Cancer	1 if respondents select cervical cancer screening from the seven options provided in the survey question, and 0 otherwise
Financial Literacy	Average score of corrects answers from three financial literacy questions (Appendix A)
Financial Education	1 if received compulsory financial education at school, and 0 otherwise
Age	Age of respondents
University education	1 if respondents have a university degree or higher, and 0 otherwise
Married	1 if married, and 0 otherwise
Divorced	1 if divorced, and 0 otherwise
Children	1 if respondents have at least one child, and 0 otherwise
Household size	The number of people living in the household
Unemployed	1 if unemployed, and 0 otherwise
Household income	Annual earned income before taxes and with bonuses of entire household in 2010 (JPY)
Log of household income	Log (household income)
Household Asset	The balanced amount of financial assets (savings, stocks, insurance, etc.) of entire household (JPY)
Log of household Asset	Log (household asset)
Current Smoker	1 if respondents smoke (occasionally–more than one pack a day), and 0 if they do not smoke (never smoke, hardly smoke, or already quit smoking)
Current drinker	1 if respondents drink (sometimes-5 cans a day), and 0 if they do not drink (do not drink at all or hardly drink)
Frequent gambler	1 if frequent gambler (once a week or more), and 0 otherwise
Myopic view of the future	1 if agree/completely agree with “Since the future is uncertain, it is a waste to think about it”, and 0 otherwise
Current Level of happiness	Percentage score from the question “Overall, how happy would you say you are currently?”
Anxiety about health	1 if agree/completely agree with “I have anxiety about my health”, and 0 otherwise
Poor Health Status	1 if current health status as poor, and 0 otherwise

### Methods

The association between breast and cervical cancer screening and the main explanatory variables is analyzed using the probit regression technique. Additional control variables are systematically introduced into the models. In the initial model (Model 1.1), demographic characteristics are included. Risky health behaviors and the future myopic view are incorporated in Models 1.2 and 1.3, respectively. Finally, the current level of happiness, anxiety about health, and health status are included in Model 1.4. This sequential approach is similarly applied to Models 2, 3, 4, 5, and 6. Impacts of financial literacy and financial education on breast and cervical cancer screening are examined independently using Eqs 1 and 2, and jointly using Eq 3.

$${\;Y}_{i}=X_{i}\beta +\varepsilon_{i\;}$$

$$Y_{i}={{FinLit}_{i}+X}_{i}\beta +\varepsilon_{i}$$ (1)

$$Y_{i}={{FinEduc}_{i}+X}_{i}\beta +\epsilon_{i}$$ (2)

$$Y_{i}={FinLit}_{i}+{FinEduc}_{i}+X_{i}\beta +\varepsilon_{i}$$ (3)

where *Y*_*i*_ represents the preventive cancer screening (either breast cancer or cervical cancer) of the respondent, FinLit denotes financial literacy, FinEduc represents financial education, and *X*_*i*_ is a vector of other explanatory variables. The complete specifications of the models are shown in Eqs 4, 5, 6, and 7. To assess the presence of multicollinearity in the models, we conduct correlation and variance inflation factor (VIF) tests. The VIF test results indicate no evidence of multicollinearity among the model variables. The correlation analysis and VIF test results are available upon request.

The specific forms of the probit regression models are as follows:

$$Y_{i}=\beta_{0}+\beta_{1}{Finlit}_{i}+\beta_{2}{Age}_{i}+\beta_{3}{Marriage}_{i}+\beta_{4}{Divorce}_{i}+\beta_{5}{Childreen}_{i}+\beta_{6}{HHSize}_{i}+\beta_{7}{UniEduc}_{i}+\beta_{8}{Unemployed}_{i}+\beta_{9}{logHHincome}_{i}+\beta_{10}{LogHHAsset}_{i}++\varepsilon_{i}$$ (4)

$$Y_{i}=\beta_{0}+\beta_{1}{Finlit}_{i}+\beta_{2}{Age}_{i}+\beta_{3}{Marriage}_{i}+\beta_{4}{Divorce}_{i}+\beta_{5}{Childdreen}_{i}+\beta_{6}{HHSize}_{i}+\beta_{7}{UniEduc}_{i}+\beta_{8}{Unemployed}_{i}+\beta_{9}{logHHincome}_{i}+\beta_{10}{LogHHAsset}_{i}+\beta_{11}{Current\;Smoker}_{i}+\beta_{12}{Current\;Drinker}_{i}+\beta_{13}{Frequent\;Gambler}_{i}+\varepsilon_{i}$$ (5)

$$Y_{i}=\beta_{0}+\beta_{1}{Finlit}_{i}+\beta_{2}{Age}_{i}+\beta_{3}{Marriage}_{i}+\beta_{4}{Divorce}_{i}+\beta_{5}{Childreen}_{i}+\beta_{6}{HHSize}_{i}+\beta_{7}{UniEduc}_{i}+\beta_{8}{Unemployed}_{i}+\beta_{9}{logHHincome}_{i}+\beta_{10}{LogHHAsset}_{i}+\beta_{11}{Current\;Smoker}_{i}+\beta_{12}{Current\;Drinker}_{i}+\beta_{13}{Frequent\;Gambler}_{i}+\beta_{14}{Futurre\;MyopicView}_{i}+\varepsilon_{i}$$ (6)

$$Y_{i}=\beta_{0}+\beta_{1}{Finlit}_{i}+\beta_{2}{FinEduc}_{i}+\beta_{3}{Age}_{i}+\beta_{4}{Marriage}_{i}+\beta_{5}{Divorce}_{i}+\beta_{6}{Childreen}_{i}+\beta_{7}{HHSize}_{i}+\beta_{8}{UniEduc}_{i}+\beta_{9}{Unemployed}_{i}+\beta_{10}{logHHincome}_{i}+\beta_{11}{LogHHAsset}_{i}+\beta_{12}{Current\;Smoker}_{i}+\beta_{13}{Current\;Drinker}_{i}+\beta_{14}{Frequent\;Gambler}_{i}+\beta_{15}{Futurre\;MyopicView}_{i}+\beta_{16}{Happiness\;Level}_{i}+\beta_{17}{Health\;Anxiety}_{i}+\beta_{18}{Health\;Status}_{i}+\varepsilon_{i}$$ (7)

### Ethical statement

This research project does not involve human experimentation. Instead, it utilizes socio-economic data. All data used in this study are sourced from third-party open access repositories. Therefore, authors do not require ethical approval for the use of these data in this study.

## Results

### Descriptive statistics

Table 2 presents descriptive statistics of the model variables. Approximately 34% of the Japanese female population participates in breast cancer screening, whereas 36.9% participate in cervical cancer screening. The results reveal a moderate average level of financial literacy, with a score of 0.528. Approximately 15.4% of the respondents receive financial education during elementary school. Respondents’ average age is 49.76 years. Approximately 80.4% of the respondents are married, with an average of one child and a household size of four. Minimal levels of divorce and unemployment are observed, with averages of 5.3% and 1.7%, respectively. The mean annual household income is 6,318,970 million yen, and the average value of household assets is 13,733,372 million yen. Approximately 15.7% of respondents have a university degree. Regarding risky health behaviors, 10.6% of respondents engage in intensive smoking, 29.3% engage in heavy drinking, and 4% are frequent gamblers. In terms of psychological factors, 98.9% of the respondents are satisfied with their current health position, 65.5% are satisfied with their current life situation, 39.2% express anxiety about later life, and approximately 16.3% have a myopic view of life.

**Table 2:** Descriptive statistics.

Variable	Mean	Std. Dev.	Min	Max
Breast cancer	0.340	0.474	0	1
Cervical Cancer	0.369	0.483	0	1
Financial Literacy	0.528	0.339	0	1
Financial Education	0.154	0.361	0	1
Age	49.765	12.352	21	77
University Education	0.157	0.364	0	1
Married	0.804	0.397	0	1
Divorced	0.053	0.225	0	1
Child	0.852	0.355	0	1
Household Size	3.514	1.433	1	11
Unemployed	0.017	0.131	0	1
Household Income	6,318,970.5	3720162	1,000,000	20,000,000
Log Household Income	1.45	0.418	0	2.485
Household Asset	13,733,372	17,806,374	2,500,000	100,000,000
Log of Household Asset	1.056	0.781	0	2.303
Current Smokers	0.106	0.308	0	1
Current Drinkers	0.293	0.455	0	1
Frequent Gambler	0.04	0.196	0	1
Future Myopic View	0.163	0.369	0	1
Level of Happiness	0.655	0.178	0	1
Anxiety of Life	0.392	0.488	0	1
Health Status	0.984	0.126	0	1
**Number of Observations: 1,729**				

The results presented in Tables 3–6 reveal variations in the likelihood of respondents undergoing breast or cervical cancer screening based on specific attributes, including age, financial literacy, and financial education. Tables 3 and 4 highlight disparities in screening based on age. The results in Table 3 suggest that breast cancer screening participation peaks at the 40–49 age group and then gradually declines. While the probability of not being screened is high across all age groups, the results in Table 4 indicate a greater participation in cervical cancer screening among respondents aged 20–29 (27.08%). The trajectory of cervical cancer screening with age is similar to that of breast cancer screening, with a turning point in the 40–49 age group. Tables 5 and 6 present the distribution of breast cancer and cervical cancer screening by financial literacy and financial education, respectively. Table 5 reveals significant disparities in breast cancer screening participation owing to the respondents’ levels of financial literacy and financial education. Similarly, Table 6 shows that the probability of undergoing cervical cancer screening is greater for those with financial education. However, a minimal variation is observed in cervical cancer screening with financial literacy.

**Table 3:** Distribution of breast cancer screening by age group.

Breast Cancer Screening	Age Group					
	20–29	30–39	40–49	50–59	60	Total
0	93	232	270	259	287	1141
	96.88%	81.98%	57.82%	59.40%	64.21%	65.99%
1	3	51	197	177	160	588
	3.12%	18.02%	42.18%	40.60%	35.79%	34.01%
Total	96	283	467	436	447	1729
	100%	100%	100%	100%	100%	100%
	F = 25.3***					

*** p<0.01

** p<0.05

* p<0.1.

**Table 4:** Distribution of cervical cancer screening by age group.

Cervical Cancer Screening	Age Group					
	20–29	30–39	40–49	50–59	60	Total
**0**	70	171	271	263	316	1091
	72.92%	60.42%	58.03%	60.32%	70.69%	63.10%
**1**	26	112	196	173	131	638
	27.08%	39.58%	41.97%	39.68%	29.31%	36.90%
**Total**	96	283	467	436	447	1729
	100%	100%	100%	100%	100%	100%
	**F = 2.69** **					

*** p<0.01

** p<0.05

* p<0.1.

**Table 5:** Distribution of breast cancer screening by financial literacy and financial education.

Breast Cancer Screening	Financial Literacy		Financial Education		
	Score <0.50	Score ≥0.50	No	Yes	Total
**0**	545	596	986	155	1141
	70.87%	62.08%	67.44%	58.05%	65.99%
**1**	224	364	476	112	588
	29.13%	37.92%	32.56%	41.95%	34.01%
**Total**	769	960	1462	267	1729
	100%	100%	100%	100%	100%
	T = -3.84***		T = -2.98**		

*** p<0.01

** p<0.05

* p<0.1.

**Table 6:** Distribution of cervical cancer screening by financial literacy and financial education.

Cervical Cancer Screening	Financial Literacy		Financial Education		
	Score <0.50	Score ≥0.50	No	Yes	Total
**0**	488	603	950	141	1091
	63.46%	62.81%	64.98%	52.81%	63.10%
**1**	281	357	512	126	638
	36.54%	37.19%	35.02%	47.19%	36.90%
**Total**	769	960	1462	267	1729
	100%	100%	100%	100%	100%
	T = -0.60		T = -3.80***		

*** p<0.01

** p<0.05

* p<0.1.

### Regression results

Table 7 presents the results of the regression models that illustrate the association between the probability of breast cancer screening and cervical cancer screening and financial education. The results indicate a positive relationship between financial education and the uptake of both breast and cervical cancer screenings. Financial education significantly contributes to the likelihood that females would be screened for breast and cervical cancers in all models, consistent with our hypothesis. However, the degree of statistical significance is more pronounced for cervical cancer screening (1%) than for breast cancer screening at the 10% significance level. Table 8 presents the results using financial literacy as the main independent variable. The results in Table 8 show a positive association between financial literacy and breast cancer screening, with varying significance levels across the models. Conversely, the relationship between financial literacy and cervical cancer is not statistically significant. Lastly, Table 9 presents the combined financial education and financial literacy results. Overall, the joint results of financial education and financial literacy with breast and cervical cancer screening are consistent in terms of signs and significance levels in all models.

**Table 7:** Results of probit model regression with financial education as the main explanatory variable.

Variables	Breast Cancer				Cervical Cancer			
	Model 1.1	Model 1.2	Model 1.3	Model 1.4	Model 2.1	Model 2.2	Model 2.3	Model 2.4
**Financial Education**	0.155* (0.087)	0.15* (0.088)	0.152* (0.088)	0.148* (0.088)	0.317*** (0.087)	0.316*** (0.088)	0.317*** (0.088)	0.312*** (0.088)

Age	0.011***	0.01***	0.01***	0.009***	-0.011***	-0.013***	-0.013***	-0.014***
	(0.003)	(0.003)	(0.003)	(0.003)	(0.003)	(0.003)	(0.003)	(0.003)
Married	0.234**	0.224*	0.224*	0.217*	0.366***	0.367***	0.367***	0.364***
	(0.117)	(0.119)	(0.119)	(0.119)	(0.122)	(0.123)	(0.123)	(0.124)
Divorced	0.541***	0.606***	0.604***	0.59***	0.68***	0.746***	0.745***	0.728***
	(0.173)	(0.176)	(0.176)	(0.176)	(0.175)	(0.178)	(0.178)	(0.178)
Child	0.108	0.124	0.123	0.13	0.068	0.084	0.084	0.095
	(0.117)	(0.119)	(0.119)	(0.119)	(0.12)	(0.121)	(0.121)	(0.122)
Household Size	-0.017	-0.011	-0.011	-0.012	0.015	0.02	0.02	0.019
	(0.026)	(0.026)	(0.026)	(0.026)	(0.026)	(0.026)	(0.026)	(0.026)
University Education	0.215** (0.09)	0.168* (0.091)	0.169* (0.091)	0.169* (0.091)	0.182** (0.088)	0.143 (0.089)	0.144 (0.089)	0.145 (0.089)

Unemployed	-0.068	-0.003	-0.008	-0.008	-0.283	-0.213	-0.215	-0.217
	(0.268)	(0.273)	(0.273)	(0.275)	(0.264)	(0.273)	(0.274)	(0.276)
Log of Household Income	0.285*** (0.089)	0.284*** (0.091)	0.285*** (0.091)	0.277*** (0.092)	0.147* (0.087)	0.144 (0.089)	0.144 (0.089)	0.138 (0.09)

Log Household Asset	0.151*** (0.047)	0.137*** (0.048)	0.139*** (0.048)	0.137*** (0.049)	0.157*** (0.047)	0.145*** (0.047)	0.147*** (0.048)	0.147*** (0.048)

Current Smoker		-0.69***	-0.691***	-0.688***		-0.582***	-0.583***	-0.583***
		(0.126)	(0.126)	(0.127)		(0.113)	(0.113)	(0.114)
Current Drinker		0.027	0.028	0.031		-0.01	-0.01	-0.004
		(0.071)	(0.071)	(0.071)		(0.07)	(0.07)	(0.07)
Frequent Gambler		0.271* (0.161)	0.268* (0.161)	0.266* (0.161)		0.247 (0.157)	0.246 (0.158)	0.247 (0.158)
Future Myopic			0.063	0.06			0.042	0.038
			(0.087)	(0.087)			(0.086)	(0.086)
Happiness Level				0.149 (0.193)				0.161 (0.189)

Anxiety Level				0.065				0.124*
				(0.068)				(0.067)
Health Status				-0.166				-0.13
				(0.255)				(0.254)
Constant	-1.857***	-1.769***	-1.77***	-1.701***	-0.679***	-0.582***	-0.582***	-0.563
	(0.221)	(0.222)	(0.222)	(0.352)	(0.219)	(0.222)	(0.222)	(0.353)
Observations	1729	1729	1729	1729	1729	1729	1729	1729
Pseudo R^2^	0.041	0.056	0.057	0.057	0.034	0.046	0.046	0.048

*** p<0.01

** p<0.05

* p<a0.1.

**Table 8:** Results of probit model regression with financial literacy as the main explanatory variable.

Variables	Breast Cancer				Cervical Cancer			
	Model 3.1	Model 3.2	Model 3.3	Model 3.4	Model 4.1	Model 4.2	Model 4.3	Model 4.4
**Financial Literacy**	0.198** (0.098)	0.164* (0.099)	0.166* (0.099)	0.177* (0.099)	-0.009 (0.096)	-0.039 (0.097)	-0.038 (0.097)	-0.021 (0.097)

Age	0.011***	0.01***	0.01***	0.009***	-0.01***	-0.011***	-0.011***	-0.012***
	(0.003)	(0.003)	(0.003)	(0.003)	(0.003)	(0.003)	(0.003)	(0.003)
Married	0.237**	0.227*	0.228*	0.221*	0.377***	0.378***	0.378***	0.375***
	(0.117)	(0.119)	(0.119)	(0.119)	(0.123)	(0.124)	(0.124)	(0.125)
Divorced	0.531***	0.597***	0.595***	0.58***	0.657***	0.724***	0.723***	0.707***
	(0.173)	(0.176)	(0.176)	(0.176)	(0.176)	(0.179)	(0.179)	(0.179)
Child	0.129	0.141	0.141	0.149	0.089	0.103	0.103	0.115
	(0.117)	(0.119)	(0.119)	(0.119)	(0.12)	(0.121)	(0.121)	(0.122)
Household Size	-0.015	-0.01	-0.01	-0.01	0.014	0.019	0.019	0.018
	(0.026)	(0.026)	(0.026)	(0.027)	(0.026)	(0.026)	(0.026)	(0.026)
University Education	0.201** (0.091)	0.157* (0.091)	0.158* (0.091)	0.158* (0.091)	0.195** (0.089)	0.158* (0.09)	0.159* (0.09)	0.157* (0.09)

Unemployed	-0.083	-0.016	-0.021	-0.023	-0.276	-0.203	-0.204	-0.209
	(0.266)	(0.272)	(0.272)	(0.274)	(0.265)	(0.276)	(0.277)	(0.278)
Log of Household Income	0.268*** (0.09)	0.27*** (0.091)	0.271*** (0.091)	0.263*** (0.092)	0.146* (0.087)	0.146 (0.089)	0.146 (0.089)	0.139 (0.091)

Log of House Asset	0.142*** (0.048)	0.13*** (0.048)	0.132*** (0.049)	0.13*** (0.049)	0.162*** (0.047)	0.152*** (0.048)	0.152*** (0.048)	0.152*** (0.048)

Current Smoker		-0.682***	-0.684***	-0.68***		-0.588***	-0.589***	-0.587***
		(0.126)	(0.126)	(0.127)		(0.113)	(0.113)	(0.114)
Current Drinker		0.026	0.027	0.031		-0.006	-0.006	0
		(0.071)	(0.071)	(0.071)		(0.07)	(0.07)	(0.07)
Frequent Gambler		0.259 (0.16)	0.256 (0.161)	0.255 (0.161)		0.231 (0.156)	0.23 (0.156)	0.231 (0.156)

Future Myopic			0.062	0.06			0.032	0.028
			(0.087)	(0.087)			(0.086)	(0.086)
Happiness Level				0.152				0.162
				(0.192)				(0.188)
Anxiety Level				0.077				0.127*
				(0.068)				(0.067)
Health Status				-0.184				-0.154
				(0.26)				(0.254)
Constant	-1.917***	-1.821***	-1.822***	-1.741***	-0.71***	-0.605***	-0.606***	-0.567
	(0.222)	(0.223)	(0.223)	(0.355)	(0.219)	(0.222)	(0.222)	(0.352)
Observations	1729	1729	1729	1729	1729	1729	1729	1729
Pseudo R^2^	0.041	0.056	0.057	0.058	0.028	0.04	0.04	0.043

Robust standard errors are in parentheses

*** p<0.01

** p<0.05

* p<0.1.

**Table 9:** Results of probit model regression with both financial education and financial literacy as main explanatory variables.

Variables	Breast Cancer				Cervical Cancer			
	Model 5.1	Model 5.2	Model 5.3	Model 5.4	Model 6.1	Model 6.2	Model 6.3	Model 6.4
Financial Literacy	0.198**	0.164*	0.167*	0.177*	-0.01	-0.04	-0.039	-0.022
	(0.098)	(0.099)	(0.099)	(0.099)	(0.096)	(0.097)	(0.097)	(0.098)
Financial Education	0.155* (0.087)	0.15* (0.087)	0.152* (0.088)	0.148* (0.088)	0.317*** (0.087)	0.316*** (0.088)	0.317*** (0.088)	0.312*** (0.088)

Age	0.01***	0.009***	0.009***	0.009***	-0.011***	-0.012***	-0.013***	-0.013***
	(0.003)	(0.003)	(0.003)	(0.003)	(0.003)	(0.003)	(0.003)	(0.003)
Married	0.229*	0.219*	0.22*	0.213*	0.367***	0.368***	0.368***	0.364***
	(0.117)	(0.119)	(0.119)	(0.119)	(0.122)	(0.123)	(0.123)	(0.124)
Divorced	0.539***	0.604***	0.603***	0.587***	0.68***	0.746***	0.745***	0.729***
	(0.173)	(0.176)	(0.176)	(0.176)	(0.175)	(0.178)	(0.178)	(0.178)
Child	0.12	0.133	0.133	0.141	0.067	0.082	0.082	0.093
	(0.117)	(0.119)	(0.119)	(0.119)	(0.12)	(0.121)	(0.121)	(0.122)
Household Size	-0.015	-0.01	-0.01	-0.011	0.015	0.02	0.02	0.019
	(0.026)	(0.026)	(0.026)	(0.026)	(0.026)	(0.026)	(0.026)	(0.026)
University Education	0.195** (0.091)	0.152* (0.091)	0.153* (0.091)	0.153* (0.091)	0.183** (0.089)	0.147 (0.09)	0.148* (0.09)	0.147 (0.09)

Unemployed	-0.079	-0.013	-0.018	-0.019	-0.282	-0.211	-0.213	-0.215
	(0.267)	(0.272)	(0.272)	(0.275)	(0.264)	(0.273)	(0.274)	(0.276)
Log of Household Income	0.269*** (0.09)	0.27*** (0.091)	0.271*** (0.091)	0.263*** (0.092)	0.147* (0.088)	0.147* (0.09)	0.147* (0.089)	0.14 (0.091)

Log of Household Asset	0.138*** (0.048)	0.127*** (0.048)	0.129*** (0.049)	0.127*** (0.049)	0.158*** (0.047)	0.148*** (0.048)	0.149*** (0.048)	0.148*** (0.048)

Current Smoker		-0.679***	-0.68***	-0.677***		-0.585***	-0.585***	-0.584***
		(0.126)	(0.126)	(0.127)		(0.113)	(0.113)	(0.114)
Current Drinker		0.025	0.025	0.029		-0.009	-0.009	-0.003
		(0.071)	(0.071)	(0.071)		(0.07)	(0.07)	(0.07)
Frequent Gambler		0.267* (0.161)	0.264 (0.161)	0.262 (0.161)		0.249 (0.157)	0.247 (0.158)	0.248 (0.158)

Future Myopic			0.067	0.065			0.041	0.037
			(0.087)	(0.087)			(0.086)	(0.086)
Happiness Level				0.151				0.161
				(0.193)				(0.189)
Anxiety Level				0.075				0.123*
				(0.068)				(0.068)
Health Status				-0.173				-0.129
				(0.258)				(0.254)
Constant	-1.9***	-1.806***	-1.807***	-1.737***	-0.677***	-0.574***	-0.575***	-0.559
	(0.222)	(0.223)	(0.223)	(0.355)	(0.22)	(0.223)	(0.223)	(0.353)
Observations	1729	1729	1729	1729	1729	1729	1729	1729
Pseudo R^2^	0.043	0.058	0.058	0.059	0.034	0.046	0.046	0.048

Robust standard errors are in parentheses

*** p<0.01

** p<0.05

* p<0.1.

Most control variables exhibit relatively consistent signs and significance levels across all models and specifications (see Tables 7–9). Breast cancer screening is positively associated with age, while cervical cancer screening is negatively associated with age. Additionally, being married or divorced, having a university degree, and having a higher household income and assets are positively associated with both breast and cervical cancer screening. Smokers are less likely to undergo cancer screening than non-smokers. Interestingly, frequent gamblers are more likely to undergo breast cancer screening than cervical cancer screening. Furthermore, individuals with health anxiety are more likely to be screened for cervical cancer than for breast cancer.

## Discussion

This study aimed to explore whether financial literacy and financial education, as proxies for rational decision-making, could help address the low participation rates in breast and cervical cancer screenings among Japanese women. By applying Grossman’s [7] rational choice framework, we examined whether these financial factors influence the decision to engage in preventive health behaviors, specifically cancer screenings.

Our results show that individuals with higher levels of financial education tend to participate in both breast and cervical cancer screening. In contrast, individuals with higher financial literacy are likely to participate in breast cancer screening, whereas no significant impact is observed for cervical cancer screening. Further, our findings reveal that financial education positively influences both breast and cervical cancer screening, consistent with previous studies that suggest a positive association between financial education and preventive health behaviors [11,16]. Since cancer diagnosis usually involves enormous financial toxicity related to material and emotional burdens for both patients and their families [24], financially educated individuals with an enhanced understanding of financial concepts are likely to opt for cancer screening to prevent future financial hardships related to contracting this disease. Additionally, financially educated individuals are less likely to procrastinate on savings plans [25,26]. Therefore, as financial education measured in this study emphasizes saving behavior, we can reasonably assume that people with higher levels of financial education tend to adhere to their saving behavior and avoid any damaging effects of cancer on their financial status by taking cancer screening as a feasible prevention measure. Our study confirms that financially educated females probably have the cognitive ability to make informed health investment decisions, such as breast and cervical cancer screening.

Furthermore, our results indicate that financial literacy positively impacts breast cancer screening behavior, but not cervical cancer screening. We find that financial literacy considerably promotes breast cancer screening, consistent with our hypothesis based on Grossman’s [7] human capital model. Engaging in female cancer screening not only signifies a demand for medical care, but also reflects the increase in knowledge, confidence, and trust of women in healthcare, attributed to greater financial literacy. Thus, financially literate women are more likely to adhere to breast cancer screening, leading to potential benefits such as reduced sick time, improved productivity, higher income, and increased savings.

Additionally, we find that financial literacy is not significantly associated with cervical cancer screening. One possible explanation for the difference in the impact of financial literacy on cervical and breast cancer screening is the increased distrust of cervical cancer prevention practices, including HPV vaccination [27]. Over the last few years, due to the substantial coverage of media reports about adverse reactions after HPV vaccination, Japanese women have undoubtedly become unwilling to be vaccinated [28], which can negatively interfere with their tendency toward cervical cancer prevention activities. People with higher financial literacy consider cervical cancer screening to be not sufficiently effective, considering all the possible risks and harms incurred with screening tests. Therefore, determining the association between financial literacy and cervical cancer screening is challenging from a risk assessment perspective.

Moreover, we find that employment, marriage, higher educational level, higher household income, and assets have strong positive relationships with both breast and cervical cancer screening. Justifiably, people of high socioeconomic status tend to understand the benefits of cancer screening programs and take advantage of the available resources for the accessibility of medical facilities; thus, they are likely to use cancer screening services. These findings are consistent with previous studies [11,29–31]. However, while age has a positive and significant association with breast cancer screening, its association with cervical cancer screening is negative and significant, which is possibly due to the commonly mistaken perception that older populations are not at risk of developing cervical cancer after menopause [32]. Smoking is negatively associated with cancer screening, consistent with previous studies [11,33]. Lastly, except for a positive association between anxiety levels and cervical cancer screening we find that happiness level, myopic view about the future, anxiety about later life, and perception of health status do not significantly affect breast and cervical cancer screening.

The results of this study have important practical implications for public health initiatives and financial education programs. The findings suggest that financial literacy and education campaigns should be widely implemented to increase Japanese women’s awareness of the importance of female cancer screening, particularly cervical cancer screening. Providing comprehensive education on the safety and efficacy of the HPV vaccine and the cervical screening process, along with improved communication between healthcare providers and the public, can help restore public trust in the effectiveness of cervical cancer prevention methods. This is applicable not only in Japanese contexts, but also in other countries, especially low- and middle-income countries (LMICs), where financial literacy levels and the uptake of female cancer screening are low. According to Bruni et al. [34], only 9% and 11% had been screened for cervical cancer at least once in lower-middle-income and low-income countries. In the next 10 to 20 years, approximately 11 million women in LMICs are projected to be diagnosed with cervical cancer [35]. Meanwhile, although the incidence of breast cancer in LMICs is lower than in the rest of the world, the age-standardized mortality rates are much higher than the world average [36]. A major challenge for the high prevalence and mortality of female-specific cancers in LMICs is the lack of awareness of the necessity and importance of screening. Regarding this, our main findings imply an urgent need to consider improving financial literacy and financial education as an effective measure to increase awareness and participation in cancer screening as a result.

However, when interpreting the study’s results, we must acknowledge that our study has certain limitations. First, our findings should be explained cautiously considering the cross-sectional nature of our analysis. Second, our measures of financial education and financial literacy are based on simplified yes/no responses and a set of three questions, respectively, which can affect the comprehensiveness of the overall results. Finally, we were unable to control the potential confounding effect of health literacy and health education in our analysis due to data limitations. Although financial literacy and financial education were the primary focus, health literacy and health education also play crucial roles in influencing health behaviors, including participation in cancer screening. Future research should include these variables to provide a more comprehensive understanding of the determinants of cancer screening behaviors. Nonetheless, as this measurement method is well-established and widely adopted in the existing literature [11,37–39], this study provides convincing evidence of breast and cervical cancer screening behavior by using financial literacy and financial education as proxies for rational decision-making. Our study is the first to evaluate the association of breast and cervical cancer screening with financial education and financial literacy in Japan, which has important implications for policy considerations throughout the country. Future studies should explore these associations using a longitudinal design for a more comprehensive understanding.

## Conclusions

Our findings underscore the significant impacts of financial literacy and financial education on breast and cervical cancer screening behavior in Japan. Given the substantial link of financial literacy and financial education to rational health decision-making, we propose enhancing them as potential solutions to address the challenge of low participation rates in breast and cervical cancer screening. Japanese policymakers should consider implementing campaigns that promote financial literacy and financial education while focusing on the benefits of early detection as a viable measure against breast and cervical cancers.

## Supporting information

S1 Checklist (DOCX)

S1 Appendix (DOCX)
