# Supplementary material for: Impact of financial literacy and education on breast and cervical cancer screening participation in Japan
Source: PLoS One. 2024 Nov 13;19(11):e0313687. doi: 10.1371/journal.pone.0313687 (PMC11560013; doi:10.1371/journal.pone.0313687)
Supplement: S1 Appendix — (DOCX) [file pone.0313687.s002.docx]

**Appendix A**

1. Suppose you have 10,000 yen in a savings account, the interest rate is 2% per year, and you never withdraw money or interest payments. After 5 years, how much would you have in this account? (X ONE box)

1. More than 10,200 yen
2. Exactly 10,200 yen
3. Less than 10,200 yen
4. Do not know
5. Refuse to answer.

2. Imagine that the interest rate on your savings account is 1% per year and inflation is 2% per year. After one year, how much would you be able to buy with the money in this account? (X ONE box)

1. More than today
2. The same
3. Less than today
4. Do not know
5. Refuse to answer.

3. Please indicate whether the following statement is True or False. (X ONE box):

“Buying a company stock usually provides a better return than a stock mutual fund”

1. True
2. False
3. Do not know
4. Refuse to answer.
